# A brief history and future directions of dielectrophoretic filtration: A review

**DOI:** 10.1002/elps.202400116

**Published:** 2024-08-09

**Authors:** Mary Clare O'Donnell, Mariia Kepper, Georg R. Pesch

**Affiliations:** ^1^ School of Chemical and Bioprocess Engineering University College Dublin Dublin Ireland; ^2^ Faculty of Production Engineering University of Bremen Bremen Germany

**Keywords:** dielectrophoretic filtration, microparticle separation, selective separation, valuable material recycling

## Abstract

Dielectrophoresis (DEP) is an electrokinetic effect first studied in the early 20th century. Since then, DEP has gained significant interest in research, owing to its ability to solve particle separation problems in various industries. Dielectrophoretic filtration (DEP filtration) is a separation method using DEP to filter a wide range of microparticles, from bacterial cells to catalytic particles. DEP filtration can selectively separate particles based on size or dielectric properties, recover trapped particles and avoid common problems associated with mechanical filtration based on pore size (e.g. pressure drops and regular filter replacements). This review describes the simple beginnings of DEP filtration and how our understanding and applications for DEP filtration have progressed over time. A brief section of DEP theory as well as a note on the general outlook for DEP filtration in the future is presented. DEP filtration offers an exciting opportunity to selectively separate diverse particle mixtures. To achieve such a feat, technical challenges such as Joule Heating and low throughputs must be addressed.

**Abbreviations**
CTCs: circulating tumour cells
DEP: dielectrophoresis
DEP filtration: dielectrophoretic filtration
eDEP: electrode‐based dielectrophoresis
EP: electrophoresis
FCCS: fluid catalytic cracker slurry
iDEP: insulator‐based dielectrophoresis
LFP: lithium iron phosphate
nDEP: negative dielectrophoresis
pDEP: positive dielectrophoresis
PDMS: polydimethylsiloxane
PS: polystyrene
PVC: polyvinyl chloride
SDS–PAGE: sodium dodecylsulphate–polyacrylamide gel electrophoresis

## 1 INTRODUCTION

### 1.1 What is dielectrophoresis?

Dielectrophoresis (DEP) is an electrokinetic effect experienced by a polarisable particle suspended in an inhomogeneous electric field. When a particle is immersed in any type of electric field, it is polarised and forms a dipole. A spatially irregular electric field will cause unequal forces on each pole. This results in a net dielectrophoretic force ($F_{\mathrm{DEP}}$) on the particle [1].

**FIGURE 1:**
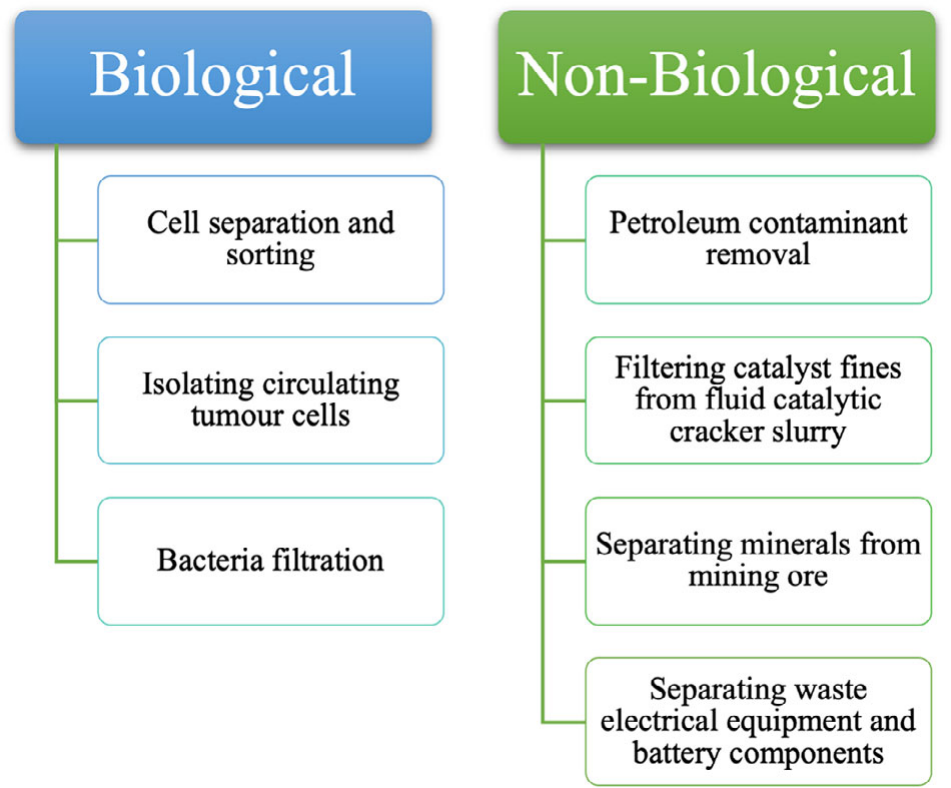
Examples of current and potential applications of dielectrophoretic separation techniques.

The **F**_DEP_ felt by a particle depends on particle size, polarisability of the particle and medium and electric field strength and non‐uniformity [2]. Particles move either towards high or low electric field regions if they are more or less polarisable than the surrounding medium (positive or negative DEP, respectively). The polarisability of a particle is determined by material, composition and surface chemistry. Usually, if a particle displays positive DEP (pDEP), it is attracted to specific regions inside a DEP device and is separated from the medium (e.g. water) and other particles. This is termed ‘particle enrichment’ or ‘particle entrapment’. Mixtures of micro and sub‐micron particles can be separated into pure particle streams using DEP by exploiting their differences in polarisability, diameter and shape. Particle mixtures can be biological (e.g. cells, bacteria or even viruses) or non‐biological (e.g. graphite, metal ore or particles from crushed electronic waste). Examples of DEP separation applications are presented in Figure 1.

Dielectrophoretic filtration (DEP filtration) is a DEP‐based particle separation technique that presents an alternative to common mechanical separation methods that exploit size (such as filtration) or density differences (such as centrifugation). DEP filtration creates an environment in which particles experience a $F_{\mathrm{DEP}}$. This results in DEP‐selective entrapment and subsequent removal of target particles from particle suspensions.

DEP filtration offers several advantages over traditional separation techniques for microparticles. DEP filtration does not suffer from filter fouling, which leads to increased pressure drops and regular filter changes [3, 4]. In addition, DEP filtration can perform material‐selective particle separation [5]. Once the electric field is switched off, trapped particles are released and can be collected from the filter, enabling the recovery of individual particle streams [6]. Consequently, it is promising for various industries ranging from biomedical to precious metal recovery, as demonstrated by an ever‐increasing number of diverse publications in the field.

Early research on DEP filtration mainly exists in the form of patents filed by those in the oil refining industry. During this period, DEP filters were of significant importance in removing impurities from hydrocarbon oils. The majority of DEP filtration research in the 20th century was dominated by the filtration of non‐biological particles from oil and adjusting filter designs to improve separation efficiencies. This continued in the 21st century, with a particular emphasis placed on removing catalyst particles from fluid catalytic cracker slurry (FCCS), a by‐product from the petrochemical refining process [7, 8, 9, 10].

Concurrently, in the early 2000s, researchers began to use DEP filtration to separate biological particles. Filter designs were inspired by those of the previous era by placing insulating media between two sheet electrodes. DEP techniques in general have proved to be a successful separation method for several biological particles in biomedical studies. For example, human cells, DNA, bacteria, mitochondria and microorganisms have all been separated using DEP methods [11]. However, most studies on, specifically the DEP filtration of biological particles, have involved yeast cells, mouse cells or bacteria. These studies are particularly useful in developing cell sorting processes in biomedical research where high throughputs are required.

This review will explore the topic of DEP filtration by analysing its origins and following how our understanding and experimental techniques have developed over the years. We will first look at the history of DEP and DEP filtration, then we will discuss basic DEP theory to provide an understanding of why DEP particles behave the way they do. Next, reviews of various studies on DEP filtration will be summarised and divided into aqueous and non‐aqueous DEP filtration. Finally, the review will end with a general outlook on the future of DEP filtration.

### 1.2 Dielectrophoresis foundations

The term DEP was first coined by Pohl in 1951 after conducting his simple yet elegant experiments, analysing the behaviour of carbon particles in a petri dish filled with di‐isopropyl ketone [12]. An inhomogeneous electric field was created by lining the dish perimeter with tin foil, acting as an outer electrode, and placing a tungsten wire inner electrode in the centre of the dish. A potential difference of 10 kV was applied across the electrodes. Graphite is conductive and attracted to regions of high electric field gradient; therefore, it experienced a **F**_DEP_ towards the sharp electrode edges. What made the particles’ behaviour unusual was that they continued to move towards the same electrode in both AC and DC conditions. In other words, the DEP force direction was independent of the electric field polarity. This is what distinguishes the phenomenon of DEP from electrophoresis (EP)—another type of electrokinetic motion. EP is the progressive movement of charged particles towards or against a (usually uniform) electric field under DC conditions [2]. In an AC field, EP results in zero net movement of a charged particle due to continuous switching of field polarity. EP is often used to separate mixtures of cells that migrate differently under such conditions. A common application of EP is the classification of protein structures depending on their lengths, called sodium dodecylsulphate–polyacrylamide gel EP (SDS PAGE) [13].

Pohl continued his research on DEP throughout the following 20 years and contributed to the field with detailed observations of cell behaviour under DEP. In 1971, Pohl and Crane carried out a series of experiments and noticed how DEP particle behaviour was affected by parameters such as electric field strength, frequency and uniformity, medium conductivity, run time and cell concentration [14]. Two platinum pin electrodes were set up to create an inhomogeneous electric field (up to 100 V_rms_), and AC frequencies of 100 Hz –10 MHz were applied. Yeast cells experienced a **F**_DEP_ and adhered to the pin electrode. Electrokinetic side effects were observed, including cell ‘stirring’ and rotation as a result of thermal hot spots created by the electrodes. This paper highlighted the complexity of DEP and how it can be affected by multiple experimental parameters [14].

Interestingly, the use of DEP to filter or separate particles has been around since 1924, with Hatfield's work considered the earliest patent in the field [15]. Hatfield developed a dielectric filtration device that could selectively separate particle constituents from a particle mixture. The filter was composed of a pair of sharp‐edged electrodes, spaced <1 mm apart, through which a slurry of metal ore suspended in nitrobenzene medium flowed [16]. If the relative permittivity of the particles was smaller than that of the medium, they would flow through the filter without being separated. If, however, their relative permittivity was larger than that of the medium, they became attracted to the electrode edges, resulting in successful particle separation. For example, Hatfield's filter recovered a conducting natural mineral, galena (lead sulphide), from a zinc blende (zinc sulphide) mixture highlighting the value of DEP separation methods in the mining industry.

## 2 THEORY

This theory section is provided to give the reader a general overview of basic DEP theory and context for the research papers reviewed later. For a more in‐depth description of DEP theory, the reader is advised to refer to popular DEP textbooks by Green and Morgan and by Pethig [1, 17]. DEP is the movement of polarisable matter in inhomogeneous electric fields. To achieve inhomogeneity, we can use several methods. Most commonly, electrode‐based DEP (eDEP) and insulator‐based DEP (iDEP). In eDEP, asymmetric microelectrodes are arranged in a microfluidic cell, creating an irregular electric field by electrode asymmetry. iDEP creates an irregular field by placing an insulating matrix between two electrodes to create regions of high and low electric field gradient [18].

### 2.1 DEP filtration principle

DEP filtration, which is the focus of this review, is a particular form of iDEP. The insulating material (called insulating matrix) is polarised under an electric field. This will generate local polarisation fields induced by the insulating material, resulting in local inhomogeneities in the electric field. Glass beads are a common insulating material used in DEP filtration. Using glass beads as an example, high electric field gradients are located at bead edges, whereas low electric field gradients are present at bead interstices (gaps between adjacent glass beads) [6]. Larger deviations in medium and insulating matrix permittivity result in provide greater polarisation at insulator boundaries, electric field gradients and particle trapping. Particles will accumulate at the insulator edges/interstices depending on their electric permittivity/conductivity relative to the medium and field frequency [19].

The size and morphology of insulating matrix components can affect the electric field gradient intensities at the matrix boundaries and separation efficiencies. For example, Kepper et al. examined the effect of insulating matrix type on particle trapping by comparing glass beads, sand and crushed glass [20]. At equal matrix particle diameters, crushed glass displayed the highest particle trapping followed by sand and glass beads. This suggests that insulating matrices with sharp edges result in more intense electric field gradients and greater separation efficiencies. The higher trapping demonstrated by sand compared to glass beads can be explained by their differences in surface morphology. With each insulator type, the separation efficiency increased with decreasing matrix particle size. Smaller diameters lead to reduced porosity, more regions of high electric field gradient and greater particle trapping.

**FIGURE 2:**
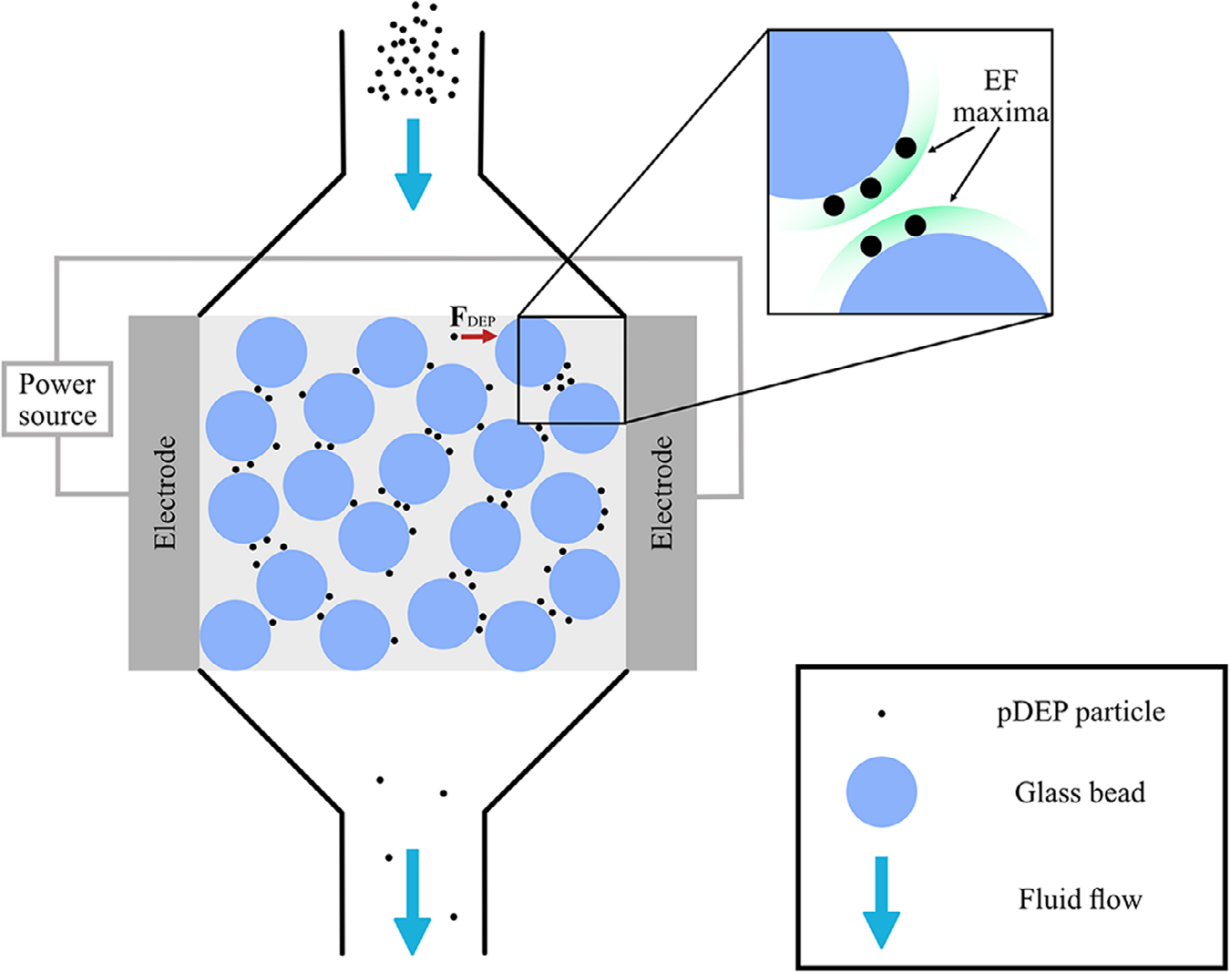
Inside a common dielectrophoretic (DEP) filter design involving two planar electrodes with glass beads as an insulating matrix (EF denotes electric field). The contact point, highlighted in green, between each glass bead is the area of high EF gradient (**F**_DEP_ is the dielectrophoretic force). For instance, 1 µm particles that experience positive DEP (pDEP) will become attracted to such contact points when an EF is applied. The electrode gap varies depending on the application of the filter. For example, the electrode gap in non‐aqueous DEP filters can be around 5 in. (127 mm) with 24 in. (305 mm) long electrodes [27]. However, the electrode gap for lab‐scale DEP filtration of cells can be as small as 1 mm [6].

DEP filtration has demonstrated its ability to separate particles at (compared to microfluidic DEP) large scales. It offers the opportunity to create a non‐uniform electric field with ease of fabrication at a macroscale. For example, Kepper et al. devised a DEP filter with glass beads packed between two stainless steel electrodes, all situated within plastic housing [20]. There are two common DEP filter designs. One consists of an outer cylindrical electrode with a coaxial inner electrode. The insulating matrix is placed between the two cylindrical electrodes [9, 21, 22, 23, 24]. The second design involves two planar electrodes with insulating material inserted in‐between [3, 4, 6, 25, 26], as illustrated in Figure 2.

To increase throughput, the flow rate can be increased. However, with greater flow rates, there is a larger drag exerted on the particle, opposing the trapping force of the filter and resulting in reduced particle trapping. If flow rate is lowered, the particle has a greater residence time in the filter and is more likely to become trapped, resulting in greater separation efficiencies. To improve trapping further, voltage can be increased as the $F_{\mathrm{DEP}}$ is proportional to the electric field strength (explained in more detail in Section 2.2).

### 2.2 Dielectrophoretic force

DEP stems from the polarisation experienced by polarisable particles within an electric field [1]. In a spatially varying electric field, the two point charges of a particle dipole experience unequal electric forces, resulting in a net **F**_DEP_. Assuming the electric field strength is constant over a small spherical particle, the **F**_DEP_ acting on that particle is described by the time‐averaged force:

(1) FDEP=34ε0εmυRefCM∇E.

[Correction added on 06 January 2025, after first online publication: $\varepsilon_{0}\varepsilon_{m}$ was included in the numerator of equations (1) and (2).]

Here  υ is particle volume, Re[*f*_CM_] is the real part of the Clausius–Mossotti factor and **E** is electric field strength amplitude. The particle velocity ($u_{\mathrm{DEP}}$) as a result of DEP is given by [28]

(2) uDEP=ε0εmυRefCM4γ∇E,

where γ is the friction factor of the particle in the medium. The real part of the Clausius–Mossotti factor (Re[fCM]) expresses the relative polarisability of the particle and determines the direction and magnitude of **F**_DEP_ and $u_{\mathrm{DEP}}$ experienced by a particle [29]. The Clausius‐Mossoti factor is calculated by

(3) $$f_{\mathrm{CM}}=\frac{\varepsilon_{p}^{*}-\varepsilon_{m}^{*}}{\varepsilon_{p}^{*}+2\varepsilon_{m}^{*}},$$

(4) $$\varepsilon^{*}=\varepsilon_{0}\varepsilon_{r}-\frac{i\sigma }{\omega }.$$

Here, $\varepsilon_{p}^{*}$ and $\varepsilon_{m}^{*}$ are the complex permittivities of the particle and medium, respectively, and $i\;=\sqrt{-1}$. $f_{\mathrm{CM}}$ depends on the relative permittivity ($\varepsilon_{r}$) and conductivity (σ) of the particle and medium as well as the angular frequency of the AC field, ω=2πf, where *f* is the frequency. The real part of Equation (3) determines the relative polarisability of the particle in the medium. The imaginary component influences the torque experienced by a particle dipole, which is constantly rotating to align with a rotating AC electric field [1]. The imaginary part is zero at lower and upper frequency limits and reaches a maximum at the Maxwell–Wagner interfacial relaxation frequency at which point electrorotation occurs.

**FIGURE 3:**
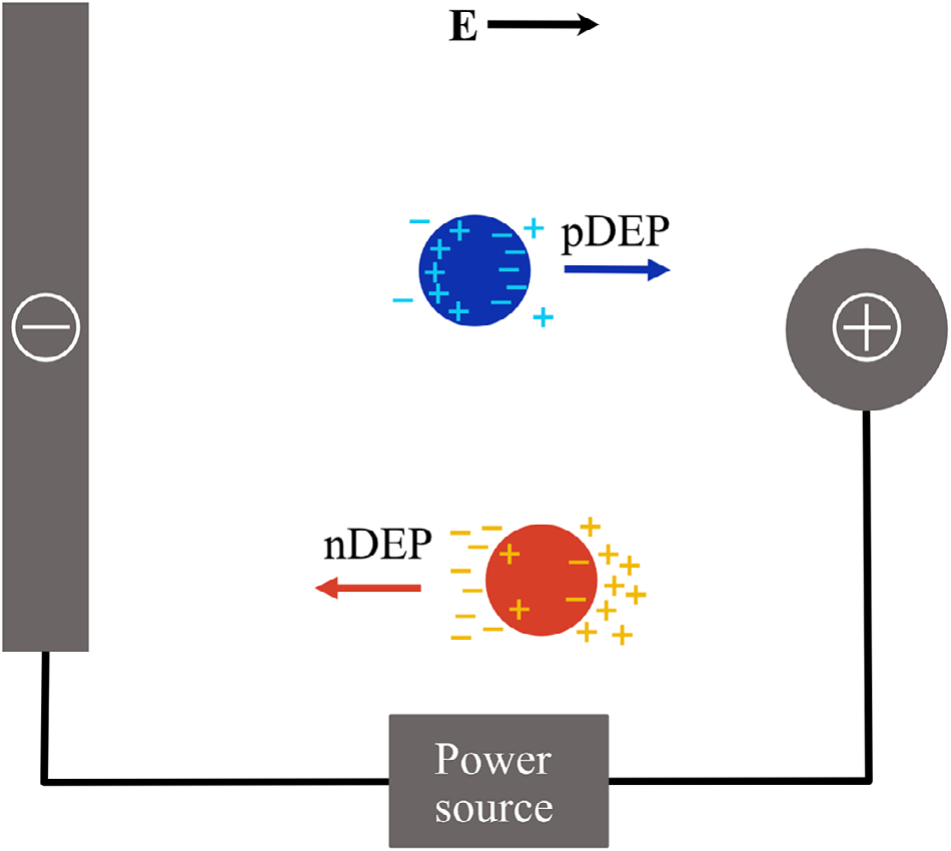
Positive DEP (pDEP) (blue) and negative DEP (nDEP) (red) particle behaviour in a non‐uniform electric field. The blue particle is more polarisable than the medium and experiences pDEP. The red particle is less polarisable than the medium and displays nDEP.

If the particle is more polarisable than the medium, Re[fCM] is positive and the particle experiences a force towards high electric field gradients. This is known as pDEP. If the particle is less polarisable than the medium, Re[fCM] is negative and the particle experiences a **F**_DEP_ towards low electric field regions. This is labelled nDEP [30]. DEP particle behaviour is illustrated in Figure 3.

### 2.3 Frequency dependence of the Clausius–Mossotti factor

Ideal dielectrics have zero conductivity; therefore, they are solely polarised by rearrangement of charged species within the dielectric. Real dielectrics, instead, possess a non‐zero conductivity and their polarisability depends on the frequency of the applied electric field (Equation (4)). Both the high and low frequency limits of Equation (3) are dependent on the particle material. At the low frequency limit of Equation (3), the polarisation of a dielectric is dominated by charge conduction as a result of the free movement of particle charges. Therefore, as frequency approaches zero, Re[fCM] is expressed in terms of conductivities, where $\sigma_{p}$ and $\sigma_{m}$ are the particle and medium conductivity, respectively [1],

(5) limω→0RefCM=σp−σmσp+2σm.

As frequency increases, conduction of free charges within a dielectric can no longer keep pace with the switching polarity of the electric field [29]. At the high frequency limit of Equation (3), a dielectric is polarised by contributions from electronic, atomic and orientational mechanisms. All of which influence the permittivity of a material. As frequency tends towards infinity, polarisability, (Re[fCM]), is described in terms of permittivity [1],

(6) limω→∞RefCM=εp−εmεp+2εm.

**FIGURE 4:**
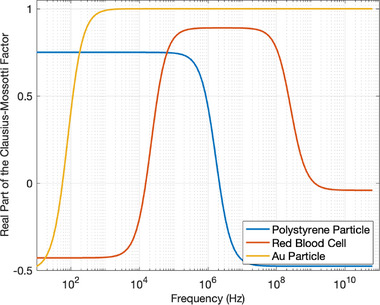
Real part of Clausius–Mossotti factor as a function of frequency for polystyrene bead, red blood cell and gold particles.

The frequency at which Re[fCM]=0 and the direction of $F_{\mathrm{DEP}}$ changes is called the crossover frequency, *f*_CO_. At this frequency, particles feel a net zero DEP force and the polarisability of particle and medium are equal (Equation (3)) [31]. A plot of Re[fCM] versus f for a range of particles (gold, polystyrene [PS] and red blood cell) suspended in water (20 µS/cm) is displayed in Figure 4. Particle properties and Re[fCM] calculations can be found in Table S1.

PS particles (1 µm) have a surface conductance of 1 nS, which contributes significantly to total particle conductivity (explained in Section 2.4) [32]. Therefore, at low frequencies, when conductivity dominates Re[fCM] in Equation (4), PS particles display positive Re[fCM] values and experience pDEP up to ∼10 kHz. PS displays a crossover frequency *f*_CO_ of about 1 MHz, at which point **F**_DEP_ = 0. Beyond this frequency, PS experiences nDEP due to its low permittivity (2.5$\varepsilon_{0}$) [32].

A single shell model was used to calculate Re[fCM] for a red blood cell [1]. This model approximates a cell as a conductive cytoplasm core encapsulated by a non‐conductive cell membrane. Both layers possess different conductivity and permittivity values. The single shell model calculates an effective complex permittivity of the cell, providing ‘smeared‐out’ bulk properties of the whole cell [33]. At frequencies between 200 Hz and ∼1.5 kHz, effective cell conductivity mainly contributes to Re[fCM], resulting in nDEP at low frequencies [34]. As frequency increases, the DEP response of a cell approaches that of a metallic particle. However, at very high frequencies, the capacitance of the membrane short circuits its high membrane resistance. As a consequence, Re[fCM] depends on the relative difference in medium and cytoplasm permittivity at frequencies beyond 10 GHz [33]. The cytoplasm in this example has slightly lower permittivity (60$\varepsilon_{0}$) than water; hence, it experiences weak nDEP.

An alternative equation (Equation (S4)) was used to calculate Re[fCM] for a gold particle to account for the electric double layer surrounding a metallic particle in aqueous solutions. In such environments, particle charges are screened by those of the electrolyte, creating an electric double layer. Therefore, at low frequencies, gold particles behave as insulators, and Re[fCM] is negative [30]. However, as frequency reaches beyond the inverse of the double layer charge time, the particle behaves as a conductor, therefore experiencing pDEP at all frequencies above the *f*_CO_ [35].

### 2.4 Double‐layer effect on insulating particles

When suspended in an aqueous solution, a double layer forms around a non‐conducting particle (e.g. PS) with a non‐zero zeta potential [36]. Maxwell–Wagner polarisation occurs at the particle and medium interface resulting in the accumulation of opposite charge surrounding the particle. A double layer (cloud of electric charge surrounding the outside of the particle) follows the particle as it moves within the medium and contributes to the particle surface conductance [31].

Total particle conductivity ($\sigma_{p,\mathrm{total}}$) is a sum of the conductivity originating from the particle bulk ($\sigma_{p,\mathrm{bulk}}$) and surface ($\sigma_{p,\mathrm{surface}}$),

(7) $$\sigma_{p,\mathrm{total}}=\sigma_{p,\mathrm{bulk}}+\sigma_{p,\mathrm{surface}},$$

where

(8) $$\sigma_{p,\mathrm{surface}}=\frac{2K_{s}}{r}.$$

$K_{s}$ is particle surface conductance and r is particle radius. $K_{s}$ is normally 1 nS for PS and dominates the overall conductivity of the particle [32]. The influence of $\sigma_{p,\mathrm{surface}}$ increases with decreasing particle size, resulting in pDEP for a wider frequency range and therefore, an increase in *f*_CO_.

### 2.5 Joule Heating, electrothermal flow and electrolysis

Joule Heating and electrothermal flow are unwanted effects that can occur in DEP experiments. High voltages in water result in an increase in thermal energy and temperature gradients [1]. Electrothermal flow occurs as a result of temperature gradients either internally (Joule Heating) or from external sources [37]. This can result in permittivity and conductivity gradients within the medium, inducing free charge density, which causes shear stress and fluid microflows within the DEP filter. Such side effects are minor in a medium with low conductivity and low electric potentials. However, at high voltage and medium conductivity, thermal convection fluid flow can arise and cause additional drag on the particle, potentially opposing DEP motion and triggering flow vortices within the filter [38]. This can result in reduced particle trapping rate and selectivity. As temperature rises, the medium can reach temperatures above its boiling point, resulting in bubble production, which disturbs particle trapping even further. The electrical power output from Joule Heating can be quantified in W/m^3^[18],

(9) $$W=\sigma_{m}E^{2}.$$

Here, $\sigma_{m}$ is the medium conductivity and **E** is the electric field strength. Equation (9) highlights the linear relationship between Joule Heating power output, electric field strength and medium conductivity. Joule Heating is particularly prevalent in aqueous DEP filtration in which high voltages are applied and a medium with appreciable conductivity is used (water). For example, Zhou et al. required a medium conductivity less than 1 S/m to prevent a temperature increase of 20 K inside their DEP filter [3].

Electrolysis is another unfavourable side effect and arises when metal electrodes are exposed to an aqueous medium inside a DEP filter. At low frequency and high voltage, electrolysis occurs at the electrode surface, resulting in gas evolution [28]. Increasing the applied AC frequency or coating electrodes with a thin insulating layer can help to prevent electrolysis [39].

### 2.6 Particle–particle interactions

Particle–particle interactions occur under DEP conditions. Each polarised particle creates its own miniature electric field as a result of its induced dipole. Particles become electrostatically attracted to each other when they are in close proximity. The ratio of inter‐particle force (**F**_PP_) and **F**_DEP_ can be described by [40]

(10) FPPFDEP∼6RefCMr3L43π/c43a.

**FIGURE 5:**
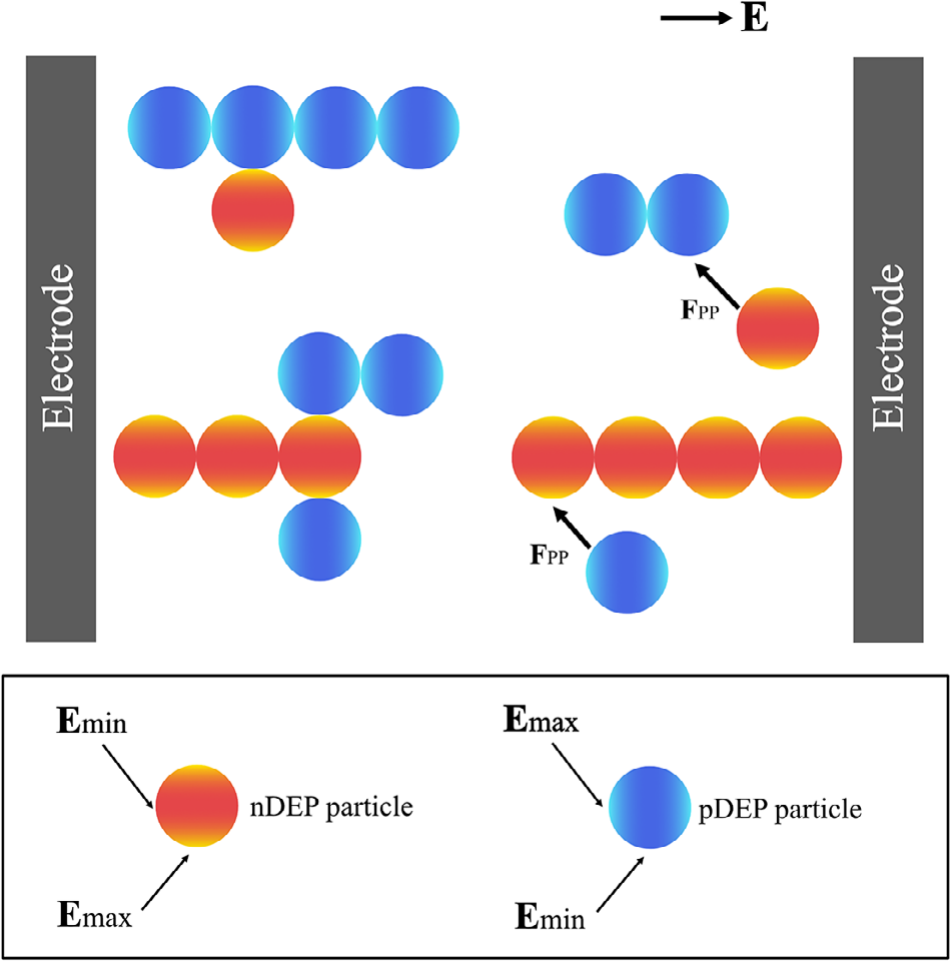
An overview of pearl chain formation [1]. **F**_PP_ is the particle–particle force and **E** is the electric field.

*r* is particle radius, *L* is electrode array dimension, *c* denotes particle number concentration and *a* is the inter‐particle distance. The particle–particle force increases with particle radius and decreasing inter‐particle distances. pDEP particles (in blue) align with their electric field maxima located at each particle pole aligned parallel with the electric field (Figure 5). However, nDEP particles (in red) arrange with their electric field maxima perpendicular to the electric field [1].

pDEP particles are attracted to electric field maxima of neighbouring pDEP particles and subsequently create a particle chain, aligning with the applied electric field. nDEP particles are attracted to the electric field minima of neighbouring nDEP particles and also form particle chains parallel with the applied electric field. pDEP particles can attach to the maxima of nDEP particles, and nDEP particles can attach to the minima of pDEP particles, forming chains perpendicular to the electric field. This process reiterates to establish long particle structures called pearl chains, which can be problematic in DEP devices. In a DEP filter with pDEP particles, pearl chains can accumulate, resulting in a short circuit that prevents further trapping [1].

## 3 DEP FILTRATION RESEARCH

DEP filtration has developed with a shifting focus over time. Throughout the 20th century (∼1950–1990), DEP filtration was commonly used to filter contaminant particles from hydrocarbon oils on an industrial scale [22, 27, 41]. Starting in 2002, an increasing focus turned towards biomedical applications [3, 6, 42, 43] using aqueous media. Aqueous DEP filtration worked as an efficient technique to selectively separate cell types [38] and non‐biological particles [5, 44].

In this review, we grouped each publication into aqueous and non‐aqueous DEP filtration research.  In non‐aqueous DEP filtration, non‐biological particles are suspended in an oil‐based medium and DC fields are usually applied. Low medium conductivity allows for high voltages, resulting in greater **F**_DEP_ and particle entrapment. Such high electric field strengths in the kV range are generated over relatively large electrode gaps, enabling industrial‐scale throughputs in non‐aqueous DEP filters.

**TABLE 1:** Comparison of dielectrophoretic filtration studies using non‐aqueous media outlined in this review.

Year	Particles	Particle size (µm)	Medium	Insulating matrix	Insulating matrix particle/pore diameter (mm)	Electric field strength (kV/m)	Throughput (mL/min)	Separation efficiency (%)	Ref.
1950	Cu	–	Hydrocarbons	Anion active resin	–	(0.1–2 kV)	8	–	[41]
1951	Laundry dirt	–	Stoddard solvent	Glass wool	–	102	38 × 10^3^	–	[49]
1968	Oil contaminants	–	Jet fuel	Polyurethane foam	394–3150 pom	1732	–	–	[48]
1975	FeS and Fe_2_O_3_	<5	Hydrocarbon oil	Glass/ceramic beads, river gravel	0.8–6.4	197–591	76 × 10^3^	97	[27]
1977	Hydrogenation catalysts	–	Non‐petroleum organic liquids	Crushed flint, marine sand, river gravel	6.4–12.8	1575	34 × 10^3^	–	[47]
1977	FeS and Fe_2_O_3_	<5	Hydrocarbons	Glass beads	3–6.4	197–787	3 × 10^5^	90	[22]
^1982^	MgO, Cu, ilmenite and PVC		Kerosene	Glass beads	3–6	39–236	–	∼80	[51]
^1982^	PVC and Cu	35–50	Kerosene + propanol	Glass beads	6	(2–8 kV)	–	∼75	[24]
^1983^	LPG pipeline dust	–	Absorption oil and jet fuel	Polyurethane foam	787 pom	39–2362	1.1 × 10^5^	96	[25]
1993	Catalyst fines	0.001–50	FCC oil	K_2_O/Na_2_O glass beads	0.8–6.4	197–787	–	100	[46]
2000	KCO_3_, KHCO_3_, KCl, MgO and CaCO_3_	0.1–100	Siloxanes, silicon containing compounds	Glass beads	1–10	79–1969	3–70	99.5	[50]
^2003^	PVC and AC dust	5–50	Tellus 37 oil	Glass beads	0.5	640−1120	0.09	65	[53]
^2011^	Glass beads, silicon and Al_2_O_3_	<10–44	Engine oil	Polypropylene mesh	0.1	(0.2–0.4 kV)	120	–	[54]
^2019^	Catalyst particles	<3–26	Conducting oil	Glass beads	1.5–5	100–600	–	60	[55]
2020	Catalyst particles	3	Conducting oil	Glass beads	3	600	–	–	[8]
2023	Catalyst particles	<3–26	FCCS	Glass beads	1.5–4	3–4	–	59	[10]
2023	Catalyst particles	<3–26	FCCS	Glass beads	4	145	–	85	[9]
^2023^	^Catalyst particles^	^<3–26^	^FCCS^	^Glass beads^	^3.5^	^167–200^	^–^	^100^	[7]

*Note*: (a) Only maximum separation efficiencies are noted, (b) diameters in inches were converted to mm and pom = pores per metre and (c) voltage is given in brackets where electrode gap dimension is not specified.

Abbreviations: FCCS, fluid catalytic cracker slurry; PVC, polyvinyl chloride.

**TABLE 2:** Comparison of dielectrophoretic filtration studies using aqueous media outlined in this review.

Year	Particles	Particle size (µm)	Medium	Insulating matrix	Insulating matrix particle/pore diameter (mm)	Voltage (kV/m)	Frequency (kHz)	Throughput (mL/min)	Separation efficiency (%)	Ref.
2002	Yeast cells	5–10	H_2_O	Glass beads	0.2	0–1	10–1000	2.5	100	[3]
2003	Yeast cells	5–10	H_2_O	Glass beads	0.2	0–1	11–1000	1	100	[38]
2005	Yeast cells	–	H_2_O	Glass beads	0.04–0.25	0–7	1000	0.002	∼75	[56]
2007	Yeast cells	–	H_2_O	Glass beads	0.1	0–100	21	0.1	75	[6]
2007	Yeast cells	–	Phosphate buffer, H_2_O	Silica beads	0.1	0–75	10—20	0.1–0.2	90	[43]
2007	Yeast cells	–	Phosphate buffer, H_2_O	Silica beads	0.1	0–100	21	0.1	75	[42]
2008	*E coli*, Listeria, Salmonella and Staphylococcus	–	H_2_O	Glass beads	0.5	0–20	10–1000	0.1	86	[4]
2008	Yeast cells	–	H_2_O	Glass beads and barium titanate	0.02–0.07	0–8	1000	0.12–0.3	80	[23]
2013	Mouse cells	–	Sucrose solution + dextrose buffer	Glass beads	0.1, 0.5	0–50	1–300	0.013–0.017	70	[26]
2014	Silica nanocapsules	0.34	Polyelectrolyte and NaAc buffer	Polyethylene filter	0.02–0.06	133	210	0.5	65	[58]
2018	Yeast cells	–	H_2_O, sodium bicarbonate buffer	Silica beads	0.1	0–150	10	1–5	83	[11]
2018	Polystyrene and yeast cells	1,–	H_2_O	Alumina‐mullite ceramic	0.1	12–24	15–30	9	100	[59]
2020	Polystyrene and graphite	0.5–4.5	H_2_O	Alumina‐mullite ceramic	0.16–0.25	12–24	1–15	1–11	100	[44]
2022	Polystyrene	2.5	H_2_O	Polypropylene mesh	(500)	110	10–65	2	79	[45]
2023	Polystyrene	0.5	H_2_O	Crushed glass, glass beads and sand	0.15–0.36	53–106	1	11	90	[20]
2024	Graphite, lithium iron phosphate	<5	H_2_O	Sand	–	53–88	15	6	90	[5]
2024	Polystyrene and *E. coli*	1, –	H_2_O	Fiberglass fibres	–	1–5	100–5000	0.004	–	[57]

*Note*: (a) Only maximum separation efficiencies are noted, (b) voltage expressed in kV amplitude/m and (c) mesh width given in brackets for insulating matrix particle/pore diameter.

**FIGURE 6:**
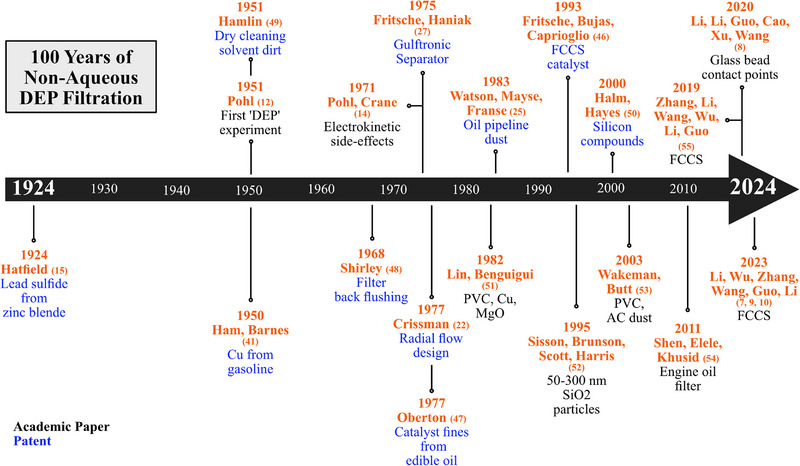
Timeline of reviewed non‐aqueous dielectrophoretic filtration studies beginning in 1924.

If DEP filtration is intended to separate biological particles (i.e. cells and bacteria), an aqueous medium is preferred, introducing new obstacles and opportunities. Water has a considerable conductivity and when high voltages are applied, excessive Joule Heating can occur. Conversely, water as a DEP filtration medium offers the ability to selectively separate a wide array of particle mixtures. For example, a binary particle mixture can be separated using a step‐wise procedure if the conductivity of water lies between the conductivity of each particle type. At low frequencies, particles with a higher conductivity than water will experience pDEP and become trapped, whereas particles with a lower conductivity than water will experience nDEP and flow through the filter [5]. Mixtures of more than two particle types can be separated in a multistep process based on their differences in *f*_CO_ or **F**_DEP_ by incrementally adjusting the field frequency or voltage [45].

### 3.1 Non‐aqueous DEP filtration

A chronological timeline of the progression of non‐aqueous DEP filtration is presented in Figure 6.

#### 3.1.1 Filtering contaminated oil

Many patents were filed in the 20th century by various oil companies focusing on the use of dielectric filters to remove metallic impurities from hydrocarbon oils. DEP filtration presented an effective, scalable and economically feasible method to remove contaminants. One of the first patents in the field focused on removing copper naphthenates from hydrocarbon oil. Naphthenates can result in gum formation, even at concentrations as low as 2 ppm, rendering gasoline unsuitable for use. To solve this problem, Ham and Barnes filed a patent in 1950 describing a dielectric filter capable of reducing copper content in gasoline [41]. The patent outlines a filter consisting of a packed bed of anion active resin, 19 mm in diameter and 445 mm long, with two platinum sheet electrodes and an applied DC potential of 100–2000 V. Gasoline with a copper content of 2 ppm flowed through the filter at 8 mL/min. The resulting concentration of copper was 0.1 ppm after filtration, demonstrating an efficient separation process based on DEP.

**FIGURE 7:**
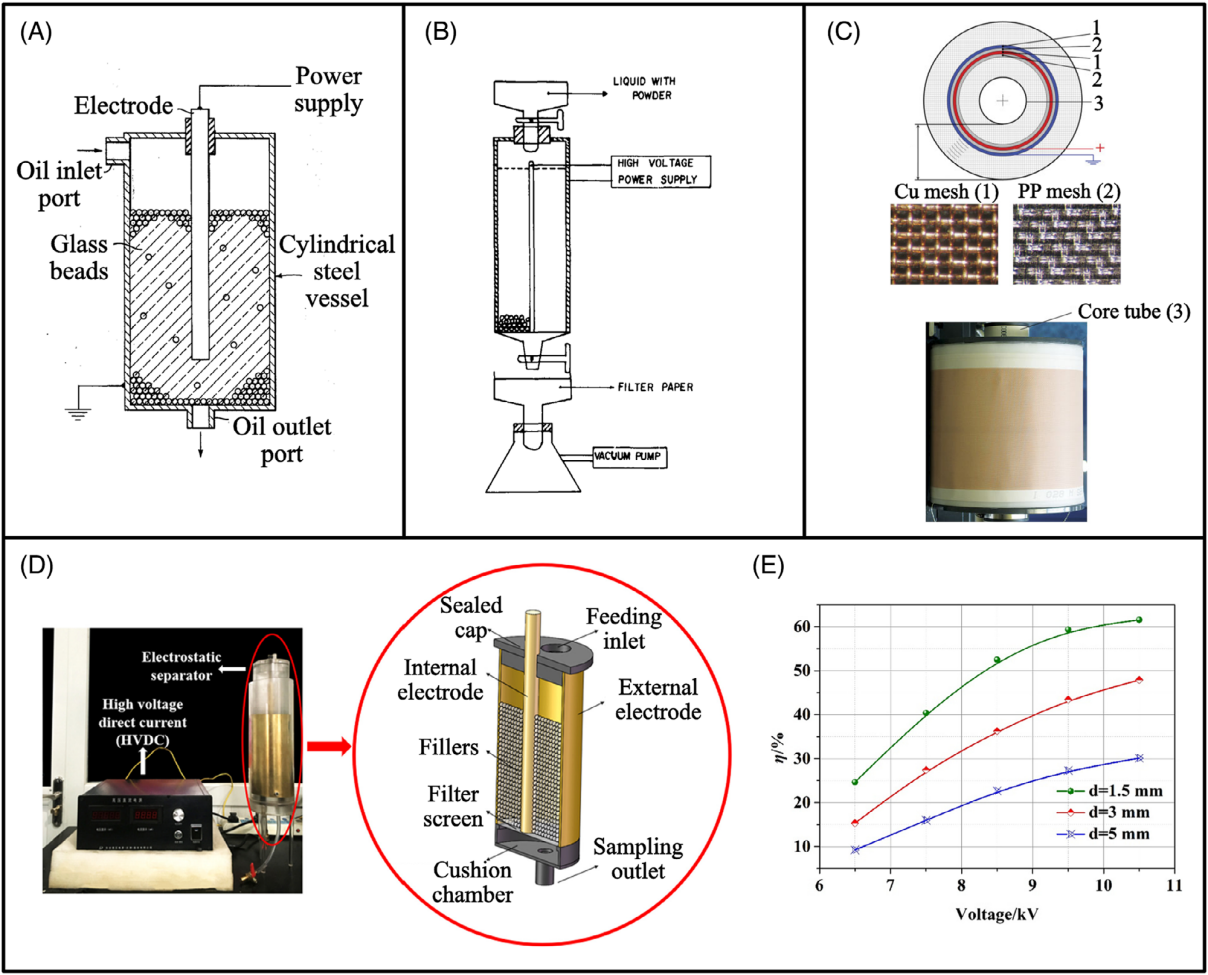
Dielectrophoresis (DEP) separator patented by Fritsche and Haniak with concentric cylindrical electrodes and glass beads as insulating material (A) [27]. DEP filter used by Lin and Benguigui to separate polyvinyl chloride (PVC) and Cu from a mixture of kerosene and isopropanol, inspired by patented DEP filters in the past (B) [24]. Used engine oil DEP filter designed by Shen et al. featuring several layers of copper and polypropylene mesh (C) [54]. DEP filter devised by Zhang et al. to separate catalyst particles from fluid catalytic cracker slurry (FCCS) (D) along with separation efficiency (η) versus voltage data (E) [55]. A general trend suggests that η increases with voltage and decreasing glass bead diameter (d). *Source*: (B) Reprinted with permission from Ref. [54] copyright (2011) John Wiley and Sons. (C) Reprinted with permission from Ref. [24] copyright (2006) Taylor and Francis (D and E) reprinted with permission from Ref. [55] copyright (2019) Elsevier.

The Gulftronic Separator sold by General Atomics is an industrial DEP filter and is still used today [21]. The original patent was written by Fritsche and Haniak of Gulf Research & Development Company in 1975 [27]. The filter described in the patent is intended to separate conductive particles (<5 µm in size) from hydrocarbon oils (Figure 7A). Using this filter design, separation efficiencies (portion of trapped particles relative to the input particle amount) of up to 97% were achieved. An example design comprised of a 150 mm ID outer electrode and 25 mm ID coaxial inner electrode, both of which were 1.8 m long. It was capable of removing even submicron particles in low concentrations of 1–3 mg/gallon (around 4–11 ppm). The patent describes a separation run operating for 75 hours at 1.5 gal/min (5.7 L/min) before completely filling up and short‐circuiting due to the formation of pearl chains.

**FIGURE 8:**
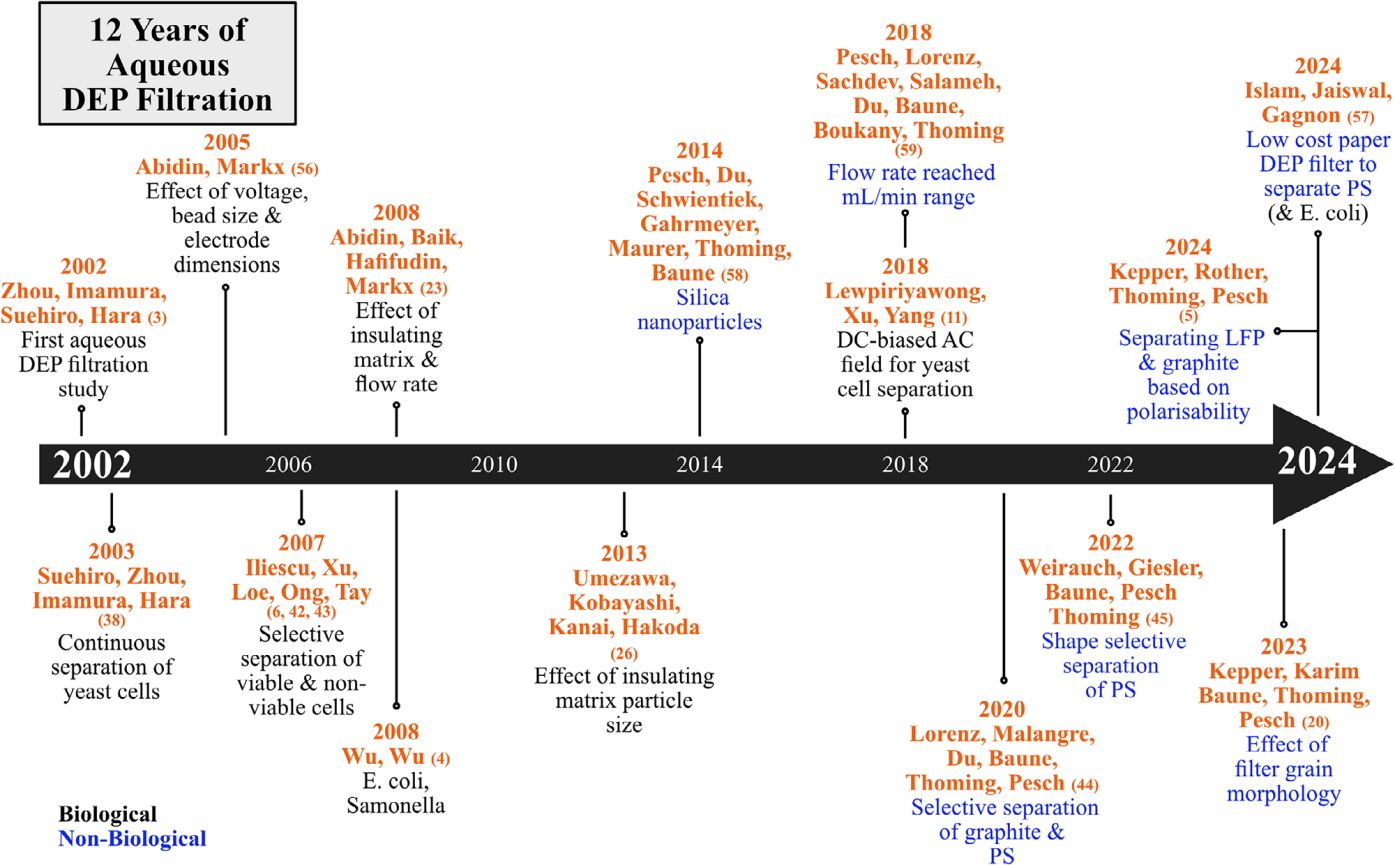
Timeline of aqueous dielectrophoretic filtration studies reviewed in this paper.

Developments in DEP filtration continued at General Atomics and in 1993, a patent by Fritsche, Bujas and Caprioglio outlined a DEP filter that removed catalyst fines (0.001–50 µm) such as alumina‐silicate from FCCS in the oil refining industry [46]. Demands grew for clearer hydrocarbon oils containing <100 ppm and sometimes <5 ppm of contaminant particles. Hence, an emphasis was placed on fine‐tuning every aspect of the DEP filters designed by General Atomics. Glass beads with higher electrical resistivity than the medium proved essential for optimal particle trapping and avoiding short circuits in the filter [27]. In this patent, glass beads, usually made from sodium oxides, were replaced with those made from a mixture of sodium oxide and potassium oxide. Potassium oxide beads displayed a more stable elevated electrical resistance, reducing the voltage requirement and improving separator lifetime. Whereas, sodium oxide beads experienced depletion of sodium ions from the bead surface and decreased electrical resistivity over long operational periods. The increased resistivity of potassium oxide compared to sodium oxide in glass beads played a noticeable role in increasing separation efficiencies up to 100%.

A DEP filter developed to separate hydrogenation catalysts from edible organic liquids, such as animal fat and vegetable oil, was patented in 1977 by Oberton of Petrolite Corporation [47]. Rather than glass beads, materials including marine sand, crushed flint and river gravel were used. A series of several concentric cylindrical electrodes were surrounded by insulating material with 20–40 kV DC applied across the system. The electrodes and insulating matrix were contained inside a 0.38 m ID cylindrical vessel, 0.76 m in height. During the hydrogenation process, oil is in contact with a Ni and Si containing catalytic material. Ni becomes suspended in the oil after hydrogenation has taken place; therefore, it requires effective removal to attain edible‐grade quality. The filter was capable of removing catalysts that were present in low concentrations of 0.01%–0.5% w/w. The filtered oil appeared clear and the resulting Ni content was between 0 and 3.4 ppm.

#### 3.1.2 Refining filter designs

DEP filter design soon became a subject of interest. In previous studies, the particle suspension commonly flowed parallel with the concentrically‐aligned electrodes. The direction of flow and its effect on particle trapping was examined by Crissman et al. from Gulf Research & Development Company who patented a DEP filter with a radial flow design in 1977 [22]. The filter removed iron sulphide and iron oxide particles (<5 µm) from crude oils using concentric stainless steel, 1.27 m long, cylindrical electrodes packed with glass beads. The outer electrode sleeve with ID 150 mm surrounded an inner electrode with ID 50 mm. The suspension entered the vessel through perforations in the inner electrode and was directed radially towards the outer electrode. The patent claimed that the radial flow design reduced linear velocity, lowering the drag exerted on particles and subsequently increasing trapping efficiency. This enabled effective filtration at greater flow rates. The radial flow filter operated at 80 gal/min (303 L/min) whilst trapping >90% of particles. However, a comparable cylindrical filter with longitudinal flow could only achieve >90% particle removal at a reduced flow rate of 20 gal/min (78 L/min). This patent demonstrates the significant impact of filter design on trapping efficiency and, in particular, the considerable advantage of incorporating a radial flow design.

In the following decade, Watson et al. of Petrolite Corporation patented a radial flow filter to remove pipeline dust contaminants present in heat absorption oil. The oil was used in a refrigerant absorption system as part of a liquefied petroleum gas recovery plant [25]. Dust particles in absorption oil result in corrosion of parts and reduced heat exchange efficiency within the refrigerant system. Therefore, an effective separation method is essential to remove such oil contaminants and ensure optimal operating conditions. The design differed to that of Crissman et al. in that multiple planar parallel electrodes were arranged horizontally within the vessel. Additionally, the direction of flow was reversed. Particle suspension entered from the perimeter and clean oil was collected in the centre. The electrodes were made from a permeable expanded metal, and polyurethane foam was placed between each electrode pair. Multiple polyurethane foam layers resulted in greater trapping compared to using only one foam layer. The device filtered a suspension with a particle concentration of 47 mg/gal (177 ppm), demonstrating 96% particle removal at 30 gal/min (114 L/min).

The backflushing mechanism is another aspect of filter design that was improved upon to increase particle recovery following filtration. After long operating periods, trapped particles can completely fill up the free space within a DEP filter. The filter is often cleared of particles by switching off the electric field and backflushing. However, this method is not always effective in removing all particles. In 1968, a patent was filed by Shirley of Petrolite Corporation describing a more effective method to remove trapped particles [48]. The corresponding DEP filter was used to remove fine particles from oils such as jet fuel. It incorporated a polyurethane foam placed between two electrodes, separated 1 in. (25.4 mm) apart. A reduction in output pressure or filtrate clarity signalled the reduced filter capacity and triggered a particle unloading procedure. Once the system detected the filter was packed full of particles, a pressurised liquid/inert gas mixture entered the vessel at 123 psi. This created hydraulic disturbances and resulted in particle agitation and effective particle removal.

#### 3.1.3 Filtering unique suspensions

To solve a very different problem, a patent was filed in 1951 by Hamlin of Hoffman Machinery Corporation [49], describing a technique of separating dirt particles from dry cleaning solvent. Glass wool was sandwiched between galvanised iron mesh electrodes placed 5 in. (127 mm) apart. A high DC voltage of 10–15 kV was applied across the electrodes. The medium was a petroleum‐based solvent known as Stoddard solvent, widely used in the dry cleaning industry. Flow rates as high as 10 gal/min (almost 38 L/min) were achieved. The patent cites the disadvantages of traditional filtration methods as the motivation for developing such filter. Mechanical filter presses present problems such as low flow rates, labour consuming cleaning and productivity losses due to regular filter replacements. Conversely, DEP filtration used for this application, achieved flow rates 20 times higher than that reachable by mechanical filtration.

DEP filtration can also be used to filter particles from non‐conducting liquids other than hydrocarbon oils. Dow Corning Corporation developed thousands of products from sealants and adhesives to silicon oils. In 2000, employees Halm and Hayes patented an ‘electric field enhanced separator’ [50]. The device was capable of removing contaminants (0.1–0100 µm in size) such as potassium carbonate and magnesium oxide from silicon‐containing liquids. The range of suspension media varied from siloxanes and silanes to silicon‐containing polymers, which were precursors for silicone resin preparation. Glass beads were packed between tubular electrodes. The entire vessel was 5.1 cm in diameter and 43 cm long. Medium temperatures during the tests ranged from 25 to 180°C with flow rates between 3 and 70 mL/min. The results demonstrated a general trend indicating the filtration efficiency increased with temperature.

#### 3.1.4 Gaining a deeper understanding of non‐aqueous DEP filtration

Following many patents filed to solve specific problems at industrial scale, several research groups began to develop a deeper understanding of the mechanisms involved in DEP filtration. In 1982, Lin and Benguigui's work expanded the range of investigated particle types to include MgO, ilmenite, polyvinyl chloride (PVC) and Cu (particle radii of 35–50 µm) [51]. Coaxial cylindrical electrodes were used. The inner electrode diameter ranged from 2 to 8 mm, highlighting the significant drop in scale compared to previous studies mentioned earlier. The behaviour of ceramic, metallic and plastic particles in kerosene was analysed. Particle yields were highest for ilmenite followed by Cu, PVC and MgO. Ilmenite and Cu experienced highest trapping due to greater permittivity values relative to kerosene. The difference in density between particle and medium is thought to play a role in particle trapping. Ilmenite has a lower permittivity yet greater density than Cu, which resulted in greater particle yields. After two filtration runs, separation efficiencies of 70%–98% were achieved. Lin and Benguigui followed up their 1982 study with another during the same year that implemented relatively conductive mediums by mixing isopropanol with kerosene in varying ratios (Figure 7B) [24]. As medium conductivity increased, Re[fCM] dropped along with **F**_DEP_ (Equation (1) and Equation (5)), resulting in reduced separation efficiency of PVC. Cu particles displayed no change of yield as the conductivity of medium increased.

Particles of interest in DEP filtration become even smaller as highlighted by a paper by Sisson et al. in 1995 [52]. In what they call a dielectric bed filtration device, silicon dioxide particles with diameters as small as 50 and 300 nm were filtered from *tert*‐amyl alcohol. The process was presented as an alternative to expensive rapid removal techniques such as centrifugation and distillation to filter ultra‐fine ceramic particles from colloidal suspensions. Glass beads were packed between stainless steel mesh electrodes in a 3 cm long cylindrical filter with a 2.5 cm diameter and flow rates up to 8.4 mL/min. With 50 nm SiO_2_ particles, outlet concentration reached 1 g/L after ∼58 min (starting at 1.8 g/L). In addition, as the temperature of the medium increased, the medium viscosity dropped resulting in reduced drag. This led to less DEP‐opposing forces and improved separation efficiencies.

Research on DEP filtration in non‐aqueous media continued to progress with Wakeman and Butt's paper in 2003, which describes a benchtop‐scale DEP filter [53]. PVC and air‐conditioning dust particles (5–50 µm in size) were separated from Tellus 37 oil and an emphasis was placed on understanding how various parameters affect the separation efficiency. Seven concentric electrodes were arranged 20 mm apart and were packed with glass beads on either side, up to a depth of 150 mm. The paper determines that highly viscous media result in large fluid drag, which opposes **F**_DEP_ and obstructs particle trapping. As the filter operation ran over time, the separation efficiency reduced due to less capture sites available. Separation efficiencies of 65% were achieved at relatively low flow rates of 0.09 mL/min.

More recently, DEP filtration of non‐biological particles in hydrocarbons has expanded to further applications. In 2011, a paper by Shen et al. describes a new concept for filtering used engine oil using DEP filtration (Figure 7C) [54]. The device was intentionally designed to filter relatively large throughputs. With high flow rates, drag forces need to be overcome by a strong **F**_DEP_. Therefore, a highly inhomogeneous electric field was created by winding alternating sheets of copper mesh and polypropylene fibres into a 5 L cylindrical filter. Glass beads, silicon and aluminium oxide (<10–44 µm in size) were tested as model contaminants in engine oil, flowing through the filter at 120 mL/min. The throughput achieved was similar to that of low‐end conventional mechanical oil filters. Although, if flow rates were increased any further, contaminant particles only weakly attach to the polypropylene mesh and drag forces would easily cause detachment, highlighting that further development is required to replace conventional engine oil filters with DEP filters.

Research on DEP filtration of catalyst particles from FCCS remains of significant importance, as demonstrated in 2019 by Zhang et al. [55]. Common methods to filter such particles include gravitational settling, mechanical filtration, centrifugation and electrostatic filtration (DEP). DEP filtration is recurrently favoured due to its ability to process large throughputs, high efficiencies and low pressure drops. Simulations and experiments were employed in this study to analyse the effect of glass bead size in a DEP filter used to separate catalytic particles from heat conducting oil (Figure 7D). Glass beads with varying diameters (1.5, 3 and 5 mm) were packed between two cylindrical electrodes with an applied DC voltage of 5–11 kV. Larger glass beads resulted in greater porosity, slower particle movement and reduced separation efficiencies. Smaller beads led to reduced porosity, improved space utilisation and less space for particles to move, which increased separation efficiencies (Figure 7E).

The same research group began to look more closely at filter design and high electric field gradient regions, the effect of contact angles between glass beads and electric field vectors at contact points in 2020 [8]. The group examined this subject using both simulation and experiment. The separation efficiency of FCCS catalyst particles suspended in oil at 15 kV DC voltage using glass beads as filtration matrix was analysed. As the angle between the electric field direction and the line of contact of two adjacent glass beads increased, the charge density at contact points decreased. Therefore, electric field strength at each contact point was at a maximum when the glass beads were arranged parallel to electric field lines. During simulation, the electric field strength at contact points between ellipsoidal glass beads was greater than that of spherical beads.

This group continued their work in 2023 by exploring the effect of catalyst particle size (<3–26 µm), particle concentration, voltage, flow rate, viscosity, temperature, FCCS composition and separation time on filtration efficiency [7, 9, 10]. Each study involved a DEP filter composed of glass beads packed between two concentric stainless steel cylindrical electrodes with an applied DC voltage. In one paper, the separation efficiency increased with higher temperatures due to the reduced medium viscosity and drag. An optimal separation temperature of 120°C was acquired. In another publication, trapping remained stable with varying catalyst concentrations and increased with separation time until filter saturation after 8 h. In addition, separation improved with increased packing heights and smaller glass beads as a result of additional contact points between glass beads and regions of high electric field strength. Separation efficiencies ranged between 59% and 100% depending on experimental parameters.

A tabulated overview of non‐aqueous DEP filtration is presented in Table 1 below.

### 3.2 Aqueous DEP filtration

A timeline of aqueous DEP filtration research is outlined in Figure 8.

#### 3.2.1 Biological particles

In contrast to non‐aqueous studies above, aqueous DEP filtration to date has been mainly carried out at relatively small scales. A study by Zhou et al. in 2002 is considered the first published in the field of aqueous DEP filtration. Here, yeast cells and cell mixtures were selectively separated from an aqueous solution [3]. The filter was comprised of glass beads placed between stainless steel electrodes and achieved trapping efficiencies of up to 99%. A wide range of voltage, 0–140 V_pp_, and frequency, 10 kHz–1 MHz, was investigated. Selective separation of viable cells from non‐viable cells was successfully demonstrated. Starting with a 50:50 mixture of viable and non‐viable cells, viable cells were trapped with a selectivity of 98% at 1 MHz after one hour of operation. Viable cells demonstrated pDEP and non‐viable cells experienced nDEP resulting in two separated particle streams (Section 2.3). The same research group continued their work the following year by analysing the effect of medium conductivity on separation efficiency [38]. The effect of Joule Heating on the particles as well as the temperature rise over time was examined. Temperature rise over time increased with medium conductivity. Joule Heating can result in a drag exerted on the suspended particles, opposing the **F**_DEP_, thereby reducing particle trapping. From the same research group, Suehiro et al. published a paper in 2003 on the continuous separation of yeast cells over a 1 h period [38]. The water temperature rise increased with medium conductivity (∼10 K at 0.2 mS/m and ∼ 50 K at 3 mS/m) as a result of Joule Heating.

**FIGURE 9:**
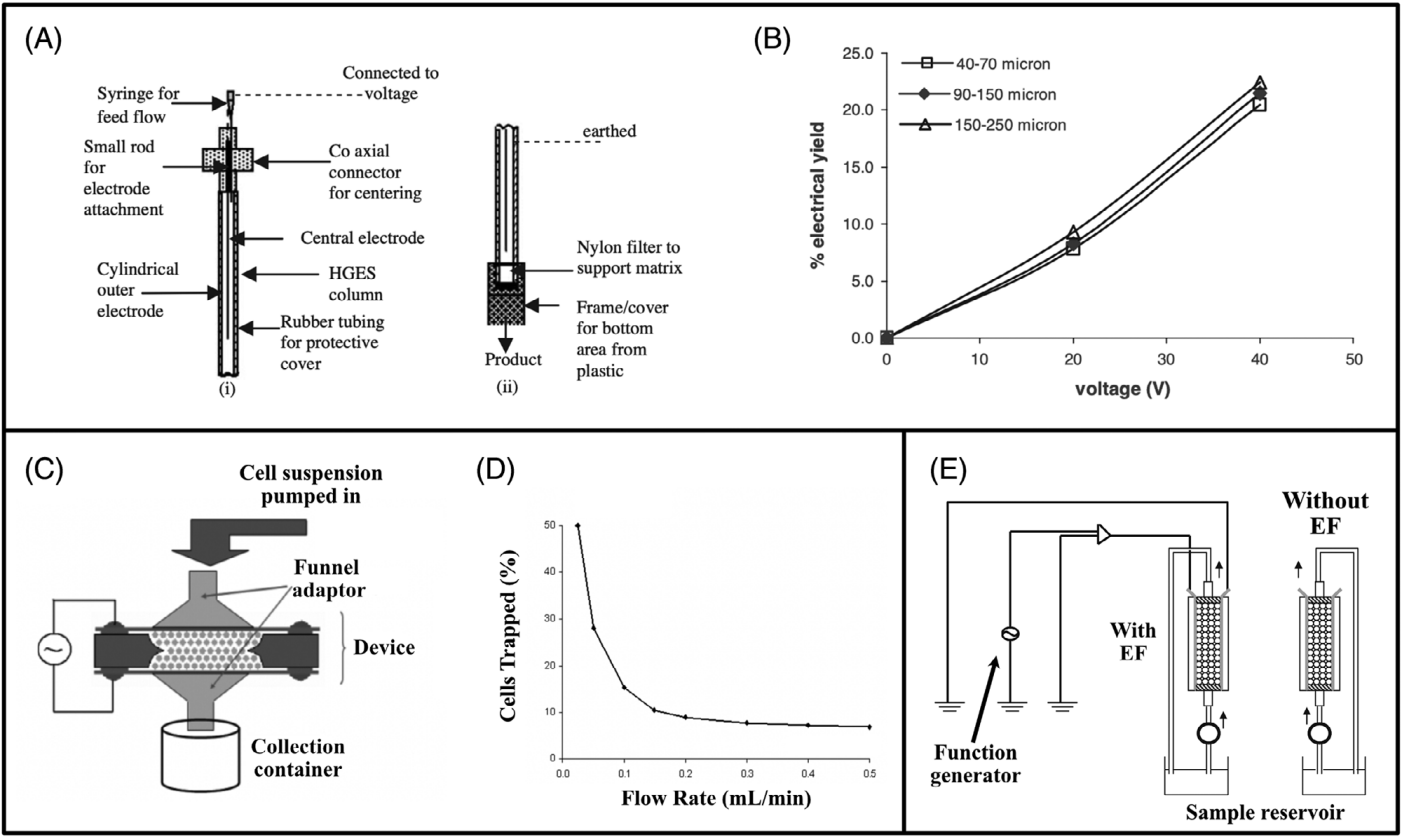
Dielectrophoretic (DEP) filter developed by Abidin and Markx featuring coaxial cylindrical electrodes with glass beads inserted in‐between (A) [56]. DEP trapping increased significantly with voltage, whereas bead size did not have a major impact on trapping (B). Iliescu et al. devised a DEP filter chip to separate yeast cell mixtures using silica beads as a filtration matrix (C) [43]. Cell trapping dropped significantly as flow rates increased (D). Schematic of a DEP filtration system devised by Wu and Wu to remove food‐borne pathogens from aqueous solutions (E) [4]. *Source*: (A and B) Reprinted with permission from Ref. [56] copyright (2005) Elsevier. (C and D) Reprinted with permission from Ref. [6] copyright (2007) John Wiley and Sons. (E) Reprinted with permission from Ref. [4] copyright (2008) John Wiley and Sons.

In 2005, Abidin and Markx systematically examined the experimental parameters of a DEP filtration column consisting of coaxial cylindrical electrodes (Figure 9A) [56]. Yeast cells were filtered from an aqueous suspension using glass beads as insulating matrix. Separation efficiency increased significantly with voltage (Figure 9B). The effect of bead size of the filter matrix on the electric field strength at their contact points was analysed using the finite element software FEMLAB. Larger beads resulted in a reduced electric field strength at each point of contact. However, the field strength further away decreased more gradually. Therefore, the trapping region was greater with larger beads, whereas the corresponding region was reduced with smaller beads. In 2008, the same group investigated the effect of voltage, insulator material and flow rate on separation efficiency [23]. Yeast cells were filtered from an aqueous suspension at 0–60 V_pp_ and flow rates of 120–300 µL/min, resulting in a mechanical trapping rate of 48%. The total separation efficiency (mechanical + DEP) increased with voltage and reached a maximum of 80% at 60 V_pp_. Two types of insulating material were tested including glass beads and barium titanate powder with a relative permittivity of 4.5 and 1600, respectively. A frequency of 1 MHz was used throughout, which suggests permittivity dictates polarisability (Section 2.3). Barium titanate resulted in the highest trapping efficiency due to its greater permittivity, creating more intense regions of high electric field gradient. As expected, the trapping rate decreased with increasing flow rates due to greater drag.

In 2007, Iliescu et al. published a series of papers using a DEP filter chip to separate yeast cells in aqueous solutions (Figure 9C) [6, 42, 43]. The filter chip had dimensions of 10 mm x 10 mm x 1 mm and was composed of silica beads packed between stainless steel mesh electrodes. Starting with flow rates of 0.1 mL/min, the filter was considered a stepping stone towards high‐throughput continuous cell capture for cell sample preparations. Trapping rates decreased with an increase in flow rate up to 0.5 mL/min (Figure 9D). Yeast cells gathered mostly at the contact zones between the beads, indicating the highest electric field gradient regions. Bubbles were formed at 200 V_pp_ due to an increase in Joule Heating and reaching the medium boiling point. The trapping efficiency increased over time due to the growing attractive force between already‐trapped cells and cells in suspension (Section 2.6). Trapping efficiencies of viable cells varied between 60% and 90%. Selective separation of viable from non‐viable cells was demonstrated by the DEP chip. Similar to studies by Zhou et al. and Suehiro et al. [3, 38], viable cells experienced pDEP, whereas non‐viable cells experienced nDEP; therefore, it was possible to separate two cell types based on their contrasting dielectric properties (Section 2.3).

The type of bio‐particles filtered in DEP devices expanded to bacteria, such as *E. coli* and *Salmonella*, both of which were investigated in a publication by Wu and Wu in 2008 [4]. Problems associated with traditional water filtration based on pore size, for example, filter blocking and regular filter changes, motivated this work on the removal of foodborne pathogens from water supplies. Two 30 mm long copper film electrodes were placed either side of an arrangement of glass beads with a relatively low applied potential difference of up to 20 V_pp_. All pathogens examined were captured on the bead surfaces, presumably experiencing pDEP, with separation efficiencies reaching 85% at 0.1 mL/min throughput. The study proved the possibility of removing bacteria from water using DEP, which could potentially reduce water filtration maintenance costs.

A study by Umezawa et al. in 2013 explored the effect of insulating matrix particle diameter on the trapping efficiency of mouse‐hybridoma 3‐2H3 cells in a DEP filter [26]. Glass beads of two sizes (500 and 100 µm) were sandwiched between two titanium plates in a device with dimensions of 50 mm x 1 mm x 1 mm (Figure 9E). Beyond 80 V_pp_, the attachment of cells reduced due to cell damage caused by elevated voltages. Separation efficiencies were higher with the 500 µm beads than the 100 µm beads at voltages up to 80 V_pp_, reaching maximum efficiencies of over 70%. This finding suggests a contradiction with improved trapping observed with smaller insulator beads (1.5 mm compared to 5 mm) by Zhang et al. [55]. However, it must be noted that a reduction in matrix particle size leads to more contact points and electric field maxima. When a cell suspension flows through these higher electric field regions, cell electroporation, cell damage and eventually reduced particle trapping may occur. Therefore, there may be a lower limit of ideal matrix particle size for the DEP filtration of cells in order to achieve optimal trapping and reduce the possibility of cell damage.

In 2018, Lewpiriyawong et al. explored the use of DC‐biased AC electric fields in a DEP filter used to separate yeast cells [11]. Separation efficiencies of 82.5% were achieved using silica beads and a Cr/Au electrode combination at 1 mL/min. The DC fields were used to control electroosmotic (EO) flow in the bead interstices with or against the inlet particle flow direction. Therefore, DC‐biased AC fields reduced the required voltage and subsequently the potential for Joule Heating, which prevented cell damage. The flow rates achieved were five times higher than a pure AC set‐up with equivalent trapping efficiencies, highlighting the advantage of incorporating DC‐biased fields. Counterintuitively, when the EO flow was in the same direction as the fluid flow, the DEP trapping was significantly higher. However, when the EO flow was against fluid flow, the capture rate was almost zero. The DC component causes EO motion along the bead's surface and slows down the net particle motion near the beads providing more time for particles to become captured.

Islam et al. published a paper on the DEP filtration of *E. coli* in 2024 [57]. A pulsed DC field (0–10 V) was applied across a paper‐based filter between two copper tape electrodes with a 1 mm electrode gap. *E coli* cells flowed through the filter at 4 µL/min for 2 min. Cells became trapped non‐uniformly inside the filter due to their immediate immobilisation upon entering. Using fluorescent intensity measurements, it was demonstrated that particle trapping increased with voltage. This paper also includes DEP filtration of non‐biological particles which is reviewed in the following section.

#### 3.2.2 Non‐biological particles

The study of non‐biological particles in aqueous DEP filters received little attention until a series of studies by Pesch et al. was published from 2014 onwards. The first of which was motivated to find an alternative, less time‐consuming and less damaging technique to filter colloidal particles in the pharmaceutical industry [58]. An emphasis was placed on increasing the throughput of the DEP filter, which involved polyethylene inserted between titanium and stainless‐steel plate electrodes. Coated silica particles (340 nm diameter) were separated from a polyelectrolyte solution at a specific flow rate of 4.12 mL/s/m^2^—several orders of magnitude greater than any other aqueous DEP filter developed in the past. A reduction in matrix pore size resulted in increased trapping efficiency up to 65%, due to shorter distances required upon particles to travel before becoming trapped, concurring with observations made by Zhang et al. on the effect of porosity [55]. Noteworthy here is that charged particles were isolated from a highly conductive polyelectrolyte solution of the same charge, a task impossible to achieve with linear electrokinetic methods.

Following on from this work, in 2018, Pesch et al. devised a DEP filter to separate PS spheres (as model particles) and yeast cells in aqueous solutions with up to almost 100% separation efficiency [59]. An increase in aqueous DEP filtration throughput was the motivation behind this study. They described their work as a stepping stone towards solving difficult separation problems with DEP, such as isolating circulating tumour cells (CTCs) from blood samples or precious metal recovery from electronic waste. An alumina ceramic insulator was sandwiched between two stainless steel electrodes at 150–300 V_rms_ and 15–30 kHz. A flow rate of 9 mL/min was achieved, facilitated by a relatively large filter cross‐sectional area of 7 mm x 29 mm. The larger filter area resulted in a lower voltage requirement than that of a PDMS microfluidic iDEP set‐up with a model porous matrix, which needed 1400 V_rms_ to achieve equivalent separation rates.

From the same research group, Lorenz et al. demonstrated for the first time the possibility to selectively separate non‐biological particles in a DEP filter at mL/min flow rates in 2020 [44]. PS and graphite were filtered using an alumina ceramic sponge placed between stainless steel electrodes, demonstrating up to 100% separation efficiencies at flow rates of 1–11 mL/min. The study examined the effects of medium conductivity, particle concentration, flow rate, filter structure size and run times on DEP separation efficiencies. At lower medium conductivities, PS experienced increased trapping efficiency due to the greater differences in particle and medium conductivity. However, as medium conductivity reached 4.2 × 10^−4^ S/m, PS trapping dropped to 10% at which point graphite displayed a trapping rate of ∼80%. This highlights the possibility to selectively separate non‐bio particles, such as PS from graphite, using aqueous DEP filtration. As run time progressed beyond 45 min, the separation efficiency decreased as a result of less available trapping sites and filter clogging. Interestingly, it was found that increasing medium pH to 8.5 aided repulsive forces between the ceramic and particles, helping to improve filter flushing and particle recovery. Joule Heating was noted as a major limitation on the medium conductivity range used in DEP filtration as it can lead to medium boiling and particle degradation.

**FIGURE 10:**
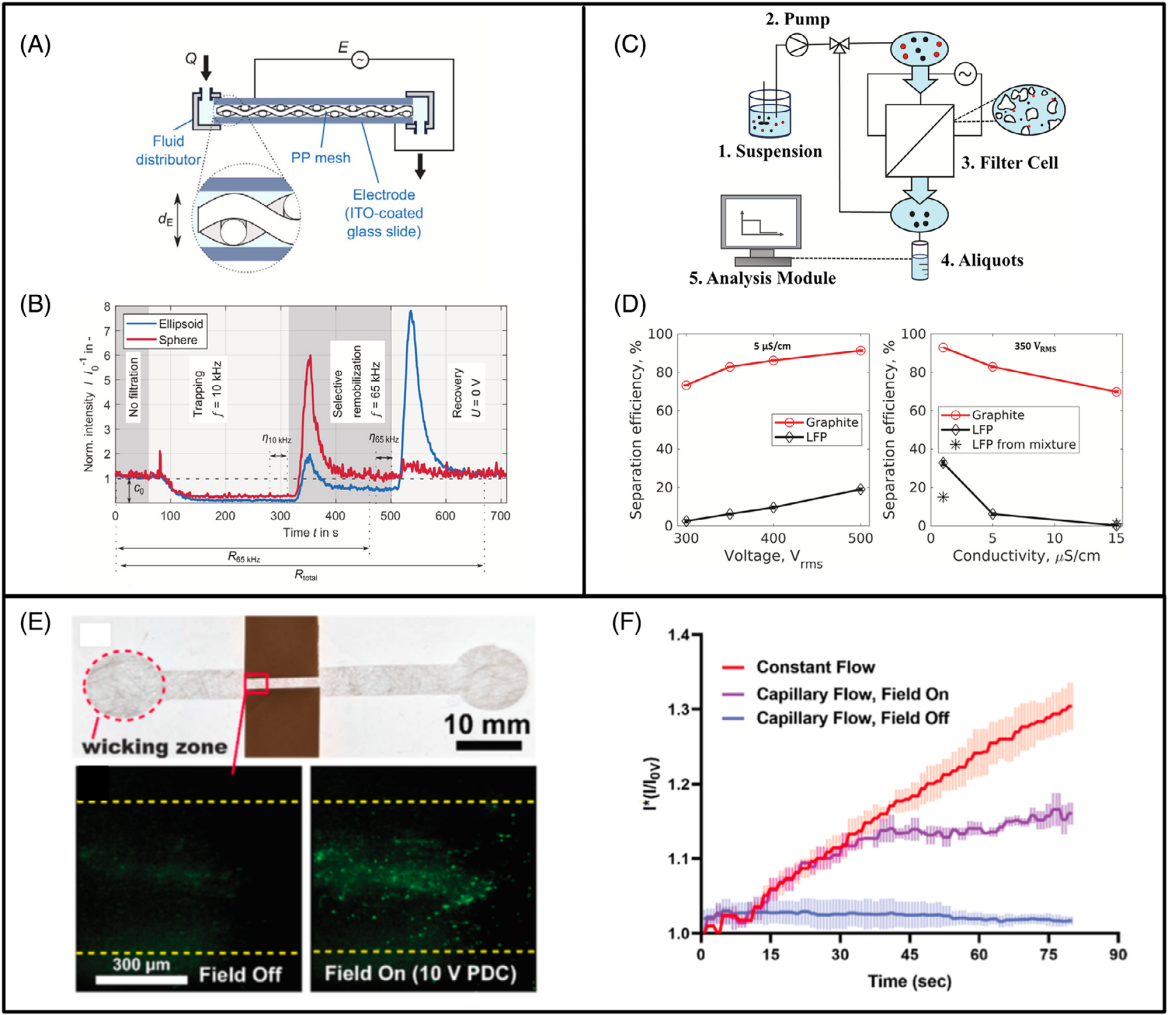
A polypropylene (PP) mesh dielectrophoresis (DEP) filter designed by Weirauch et al. captured and selectively remobilised particles based on their shape (A) [45]. At first, both spherical and ellipsoidal polystyrene particles were trapped at 10 kHz. As frequency shifted to 65 kHz, spherical particles remobilised whilst ellipsoidal particles remained inside the filter (B). The set‐up operated by Kepper et al. to filter graphite and lithium iron phosphate (LFP) using sand as an insulating matrix (C) [5]. Trapping rates of both graphite and LFP increased with voltage and dropped with medium conductivity (D). Paper‐based DEP filter developed by Islam et al. to separate fluorescently labelled PS particles (E) [57]. Trapping efficiency was highest with constant flow compared to capillary flow (F). *Source*: (A and B) Reprinted with permission from Ref. [45] published under CC‐BY license. (C and D) Reprinted with permission from Ref. [5] published under CC‐BY license. (E and F) Reprinted with permission from Ref. [57] published under CC‐BY 4.0 license.

In 2022, a DEP filter selectively separated PS particles based on shape alone in a publication by Weirauch et al. [45]. A polypropylene mesh was placed between two indium tin oxide coated glass slides resulting in a highly cost‐effective filter fabrication (Figure 10A). A binary mixture of spherical (2.5 µm diameter) and ellipsoidal (4 µm diameter) PS particles exhibited different surface conductance, therefore differences in Re[fCM] and $F_{\mathrm{DEP}}$. This enabled selective trapping of one particle type whilst the other flowed through unaffected by adjusting the AC frequency. At 10 kHz, both particle types experienced pDEP and were trapped in the filter at separation efficiencies of 70%–79%. At 65 kHz, ellipsoidal particles continued to experience pDEP and remained in the filter, whereas spherical particles felt a much weaker pDEP and, in some cases, nDEP, resulting in their release from the filter (Figure 10B). Ellipsoidal particles were subsequently discharged by switching off the applied electric field of 2.2 kV_pp_/cm. When the recovery period was increased, a higher particle yield and lower purity were achieved. Conversely, the purity increased and the yield reduced when the recovery period was shortened. Therefore, a balance needs to be struck between yield and purity depending on the application for each selective separation case.

In 2023, Kepper et al. investigated the influence of insulating matrix morphology on DEP filtration efficiency using model PS particles [20]. Sand, glass beads and crushed glass were separately incorporated between two stainless steel electrodes. A throughput of 11 mL/min along with separation efficiencies of up to 90% were achieved. A key discovery here was the relationship between the morphology of the insulating matrix and trapping efficiency. Insulating material with greater degrees of irregularity (sharper edges) resulted in higher separation efficiencies, possibly due to higher field maxima at sharp boundaries. Crushed glass demonstrated highest separation efficiency, followed by sand and glass beads, respectively. With smaller matrix particle sizes, the filter porosity is reduced, which results in improved separation efficiencies due to the shorter distances travelled by particles before entrapment. This agrees with similar findings in a study by Zhang et al. mentioned above in Section 3.1.4 [55].

In 2024, another paper from Kepper et al. examined the use of DEP filtration to separate a particle mixture of graphite and lithium iron phosphate (LFP), which are common anode and cathode battery materials, respectively [5]. Both particle types were <5 µm in size and suspended in water. Graphite possesses a higher bulk conductivity than water, resulting in pDEP, whereas uncoated LFP is less conductive than water and demonstrates nDEP. Sand was used as an insulating matrix packed between two stainless steel electrodes (Figure 10C). An electrical potential of 300–500 V_rms_ was applied at 15 kHz and trapping efficiencies reached up to 90% at a constant flow rate of 6 mL/min. Graphite trapping efficiency reduced slightly, whereas LFP trapping dropped significantly with increasing medium conductivity (Figure 10D). This was attributed to an increase in temperature with conductivity leading to unwanted thermal effects such as convection flows and disrupted DEP particle behaviour. Finally, it is worth mentioning that most Li‐ion batteries contain carbon‐coated LFP, which may exhibit pDEP behaviour, adding complexity to the separation of real battery material if both effective particle conductivities are similar. Nevertheless, selective separation based on particle size has been used to separate (usually smaller) carbon‐coated LFP from graphite using eDEP methods [60].

New types of insulating structures in DEP filters continue to be explored and modified. A study by Islam et al. in 2024 looks at the use of paper as an insulator in DEP filtration, offering cheap and scalable fabrication [57]. A fiberglass paper was placed between copper tape electrodes and PS particles were captured by DEP at 4 µL/min. An AC electric field of 2–10 V_pp_ at 100 kHz–5 MHz was applied. The particles appeared to trap well at the sharp ends of the fiberglass due to the high electric field gradients at those regions (Figure 10E). PS particle trapping was analysed using fluorescent intensity measurements, which increased with applied voltage. Fluid flow powered by capillary action in paper was considered in this study, allowing the filter to run without an external pump. However, when compared with an external constant fluid flow, capillary flow resulted in reduced particle trapping due to a decrease of particle flux entering the filter decrease over time (Figure 10F).

An overview of all reviewed articles on aqueous DEP filtration is included in Table 2.

## 4 THE FUTURE OF DEP FILTRATION: CHALLENGES AND POSSIBILITIES

A promising biological application for DEP filtration includes the separation of CTCs from normal blood cells. Gascoyne and Shim demonstrated the varied dielectric properties of cancerous and healthy cells, suggesting the possibility of separating such cell mixtures using DEP methods [61]. Cancerous cells tend to have more folds and ruffles on the cell surface compared to healthy cells, leading to differences in cell morphology and therefore, contrasting dielectric characteristics. DEP filtration of CTCs may present a useful separation technique to aid cancer research in prognosis and diagnostic processes for various cancer types.

Meanwhile, an interest in DEP filtration of non‐biological particles continues to grow. One potential application centres on the growing accumulation of electronic waste globally, in particular used lithium‐ion batteries. Hydrometallurgical methods are normally used to recover metals (e.g. Co, Mn and Ni) from battery waste, requiring large quantities of acid, several purification steps and energy [62]. Therefore, the recycling industry is in need of a more sustainable and efficient particle separation technology. Lithium metal oxide (cathode) and graphite particles possess different dielectric properties and sizes, presenting an interesting prospect of using DEP filtration to separate battery components in a clean and effective manner. The tangible possibility of using DEP filtration to recycle LIB waste in the future was highlighted by a recent study in which uncoated LFP and graphite were separated by DEP filtration based on contrasting particle polarisability [5].

This review has demonstrated the humble beginnings, progression and promising future of DEP filtration as an efficient particle separation technique placed in a wide range of industries. Water as a DEP medium opens the door to particle‐selective separation of complex biological and non‐biological mixtures. Following their comparison, aqueous DEP filtration can be inspired further by non‐aqueous research. Areas of expansion in aqueous DEP filtration include investigating broader ranges of non‐biological particles, insulating matrices and throughputs. However, Joule Heating remains a challenge, reducing particle trapping and selectivity due to electrothermal flow of the medium. This can lead to medium boiling and bubble formation, disrupting the entire DEP process. Therefore, to demonstrate superior efficiency and economic viability of DEP filtration over traditional filtration methods, key technical challenges such as low throughputs and Joule Heating must be addressed.

## CONFLICT OF INTEREST STATEMENT

The authors declare no conflicts of interest.

## Supporting information

Supporting Information

## Data Availability

Data sharing is not applicable to this paper as no new data were created or analysed in this study.
