# Supplementary material for: A brief history and future directions of dielectrophoretic filtration: A review
Source: Electrophoresis. 2024 Aug 9;46(15):1112–32. doi: 10.1002/elps.202400116 (PMC12461443; doi:10.1002/elps.202400116)
Supplement: Supplementary file 1 — Supporting Information [file ELPS-46--s001.docx]

**Supplementary Information for ‘A Brief History and Future Directions of Dielectrophoretic Filtration’**

Mary Clare O’Donnell^1^, Mariia Kepper^2^, Georg Pesch^1^

^1^ University College Dublin, School of Chemical and Bioprocess Engineering, Ireland

^2^ University of Bremen, Faculty of Production Engineering, Germany

**1 Clausius Mossotti Calculations**

- 1. **Polystyrene particle**

Eq. (S1) and (S2) were used to calculate $f_{\mathrm{CM}}$ of a polystyrene (PS) particle [1]. The real part of $f_{\mathrm{CM}}$ was plotted against frequency where $\omega=2\pi f$,

$f_{\mathrm{CM}}=\frac{\varepsilon_{p}^{*}-\varepsilon_{m}^{*}}{\varepsilon_{p}^{*}+2\varepsilon_{m}^{*}}$ Eq. (S1)

$\varepsilon^{*}=\varepsilon_{0}\varepsilon_{r}-\frac{i\sigma}{\omega}$. Eq. (S2)

- 1. **Red Blood Cell**

An effective particle permittivity ($\tilde{\varepsilon}_{23}$) was calculated to account for the contrasting dielectric properties of the cell membrane (non-conductive) and cytoplasm (conductive),

$\tilde{\varepsilon}_{23}=\tilde{\varepsilon}_{2}\frac{\left[ \gamma_{12}^{3}+2\left( \frac{\tilde{\varepsilon}_{3}-\tilde{\varepsilon}_{2}}{\tilde{\varepsilon}_{3}+2\tilde{\varepsilon}_{2}} \right) \right]}{\left[ \gamma_{12}^{3}-\left( \frac{\tilde{\varepsilon}_{3}-\tilde{\varepsilon}_{2}}{\tilde{\varepsilon}_{3}+2\tilde{\varepsilon}_{2}} \right) \right]}$. Eq. (S3)

$\tilde{\varepsilon}_{2}$= membrane complex permittivity and $\tilde{\varepsilon}_{3}$=cytoplasm complex permittivity, $a_{1}$=total cell radius and $a_{2}$=cytoplasm radius and $\gamma_{12}=\frac{a_{1}}{a_{2}}$. Eq. (S3) is substituted into Eq. (S1) as $\varepsilon_{p}^{*}$ to calculate $f_{\mathrm{CM}}$ [1].

- 1. **Gold Particle**

Eq. (S4) is used to calculate the real part of $f_{\mathrm{CM}}$ for a metallic particle such as gold, [2]

$\mathrm{Re}\left[ f_{\mathrm{CM}} \right]=\frac{\Omega^{2}-2}{\Omega^{2}+4}$. Eq. (S4)

$\Omega=\frac{\omega C_{\mathrm{DL}}a}{\sigma_{m}}$ is dimensionless, $C_{\mathrm{DL}}=\varepsilon_{M}\kappa$ is the double layer capacitance per unit area, $a$= particle radius and $\sigma_{m}$=medium conductivity.

**Table S1** Dielectric values used to calculate $\mathrm{Re}[f_{\mathrm{CM}}]$ for a PS particle, red blood cell and Au particle in Fig. 5.

| $\varepsilon_{0}$ | 8.854x10^-12^ m^-3^ kg^-1^ s^4^ A^2^ |
| --- | --- |
| **Medium** | |
| $\sigma_{m}$ | 20x10^-6^ S m^-1^ |
| $\varepsilon_{m}$ | 78$\varepsilon_{0}$ |
| **PS** | |
| $\sigma_{p}$ | 2x10^-3^ S m^-1^ |
| $\varepsilon_{p}$ | 2.5$\varepsilon_{0}$ |
| **Red Blood Cell** | |
| $\sigma_{2}$ | 10^-8^ S m^-1^ |
| $\sigma_{3}$ | 0.5 S m^-1^ |
| $\varepsilon_{2}$ | 10$\varepsilon_{0}$ |
| $\varepsilon_{3}$ | 60$\varepsilon_{0}$ |
| $a_{1}$ | 2.01x10^-6^ m |
| $a_{2}$ | 2x10^-6^ m |
| **Au particle** | |
| $a$ | 5x10^-6^ m |
| $C_{\mathrm{DL}}$ | 0.1 F m^-2^ |

**References**

[1] Morgan H, Green NG. AC Electrokinetics Colloids and Nanoparticles. England: Research Studies Press; 2002.

[2] Pesch GR, Du F. A review of dielectrophoretic separation and classification of non-biological particles. Electrophoresis. 2021;42:134-52.
